# High failure rates and poor quality of life after modular non-fusion knee arthrodesis following failed total knee arthroplasty: a multicenter retrospective study of 85 patients

**DOI:** 10.1186/s42836-026-00415-5

**Published:** 2026-07-14

**Authors:** Benjamin Schlossmacher, Christoph Tietje, Vincent Lallinger, Ruediger von Eisenhart-Rothe, Igor Lazic, Florian Pohlig, Sven Hungerer, Severin Langer

**Affiliations:** 1 Klinikum Rechts Der Isar, Department of Orthopaedics and Sports Orthopaedics, Technical University of Munich, 81675 Munich, Germany; 2 Schön Klinik Harlaching, 81547 Munich, Germany; 3 BG Trauma Center, 82418 Murnau, Germany; 4 Institute for Biomechanics, Paracelsus Medical University, 5020 Salzburg, Austria

**Keywords:** Arthroplasty, Periprosthetic joint infection, Arthrodesis, Modular, Revision arthroplasty, Salvage procedure

## Abstract

**Background:**

Modular non-fusion knee arthrodesis (MKA) represents a limb-salvage option for patients with failed total knee arthroplasty (TKA) when further reconstruction is no longer feasible, most commonly due to periprosthetic joint infection (PJI) or aseptic failures. However, evidence regarding implant survival, infection control, functional outcomes, and quality of life remains limited and heterogeneous. This multicenter study aimed to evaluate clinical outcomes after MKA and identify risk factors for treatment failure.

**Material and methods:**

This retrospective multicenter cohort study included 85 adult patients from two centers who underwent MKA after failed TKA between 2003 and 2024, with a minimum follow-up of 12 months. The primary outcome was the overall implant survival, defined as no replacement of the MKA (total or partial) nor conversion to above-the-knee amputation (AKA). Secondary outcomes included all-cause revision-free and infection-free survival (following the Delphi-consensus criteria from 2013), amputation rate, functional outcome assessed through patient-reported outcome measures (PROM) by the Lower Extremity Function Score (LEFS), and quality of life measured using the EQ-5D-3L. Survival analyses were performed using Kaplan–Meier analysis and Cox regression.

**Results:**

Sixty out of eighty-five patients (70.6%) retained their modular arthrodesis until the latest follow-up, with patients requiring replacement of their MKA (17/85) or undergoing AKA (8/85) during follow-up. The median (IQR) follow-up was 35.0 (46.0) months. There was no difference in overall implant survival between septic and aseptic indications (71.2% vs. 69.2%; *p* = 0.855). Two-stage revision was associated with an improved implant survival proportion (90.9%) compared with multi-stage (67.6%; *p* = 0.042) and single-stage revision (57.7%; *p* = 0.010). Exploratory threshold analysis suggested that patients with five or more previous surgeries experienced substantially higher failure rates. Aseptic indications, particularly painful TKA without structural failure, were associated with significantly higher amputation rates than septic cases. Among patients available for PROM assessment, functional outcomes remained poor, with a mean LEFS of 21.6/80, and quality of life was substantially reduced (mean EQ-5D-3L index 0.612), with no significant differences across subgroups.

**Conclusion:**

MKA following failed TKA is associated with only moderate implant survival and poor functional outcomes and quality of life. Especially painful TKAs carry an unexpectedly high risk of subsequent amputation, while extensive prior surgical history is a major predictor of failure. Two-stage revision was associated with improved implant survival; however, given the retrospective design and potential selection bias, causal conclusions cannot be drawn. Careful patient selection and thorough preoperative counseling remain essential. Further studies should focus on the comparison of MKA with AKA, as well as with additional revision total knee arthroplasty when feasible.

## Introduction

Total knee arthroplasty (TKA) is a highly successful procedure. However, failure can still occur, most commonly due to periprosthetic joint infection (PJI) or aseptic loosening. The infection rate following primary TKA ranges from 1 to 3% but increases substantially in high-risk patients [1–3]. Despite advances in surgical techniques and antimicrobial strategies, PJI remains a devastating complication associated with high morbidity, an impaired quality of life, and a significant socioeconomic burden [4–7].

Although it is considered the gold standard for chronic PJI, two-stage revision arthroplasty is associated with reinfection rates of up to 25%. This often leads to progressive bone loss and extensor mechanism failure, which preclude further reconstruction and necessitate salvage strategies [8–10]. When further revision TKA is no longer feasible, knee arthrodesis and above-the-knee amputation (AKA) constitute the main salvage options, with the choice reflecting a balance between limb preservation, infection control, and functional expectations. Knee arthrodesis is frequently chosen when patients decline amputation or when maintaining a stable, weight-bearing limb is deemed necessary for mobility and prosthetic fitting after amputation is unlikely. While osseous fusion of the knee has been a viable option for decades and has been thoroughly investigated, modular knee arthrodesis (MKA) is a relatively novel procedure, and the results remain scarce and heterogeneous. Previous outcomes have been reported to show only moderate success with substantial rates of reinfection and revision. A systematic review showed an overall revision rate of 18.6% with infection rates of 17.2% [11].

Clear recommendations are lacking, and guidance on choosing arthrodesis versus amputation remains insufficient.

Therefore, this multi-center retrospective study aimed to evaluate implant survival, infection control, revision and amputation rates, functional outcomes, and quality of life following non-fusion MKA after failed TKA, and to identify risk factors for treatment failure.

## Material and methods

**Fig. 1:**
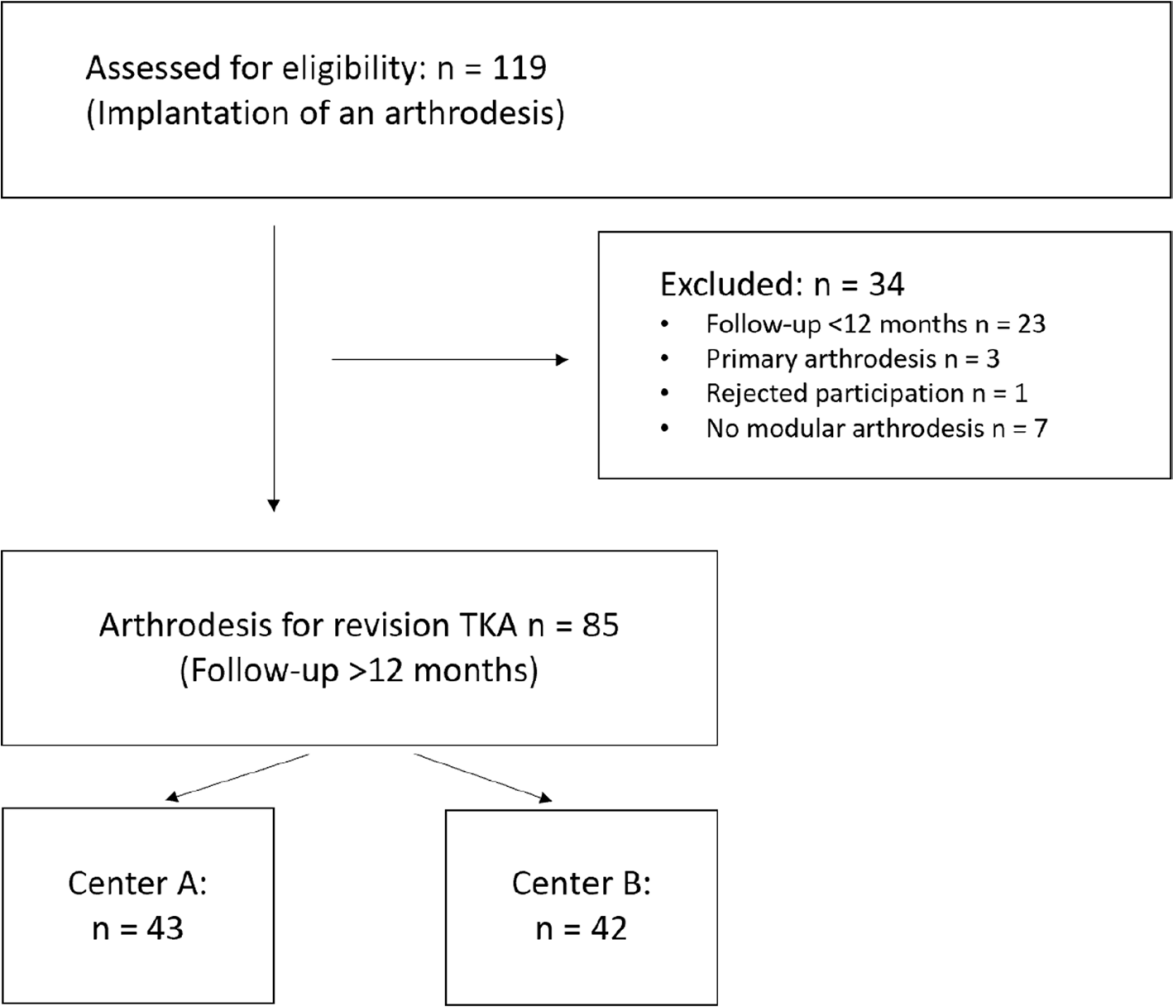
Flowchart of patient inclusion. TKA = total knee arthroplasty

We conducted a retrospective observational cohort study on a total of 119 patients undergoing arthrodesis of their knee joint between 2003 and 2024 at two independent high-volume arthroplasty centers. Eighty-five patients met the final inclusion criteria (> 18 years at surgery, > 12 months follow-up, modular non-fusion arthrodesis after failed TKA) (Fig. 1). Indications for the implantation of an arthrodesis were either an uncontrollable periprosthetic joint infection, defined following the EBJIS criteria [12], or aseptic causes such as irreparable extensor mechanism failure with or without aseptic loosening of the TKA.

Patient demographics, comorbidities (Charlson Comorbidity Index with age inclusion), PJI characteristics, implant data, functional outcome, and general quality of life were documented.

### Center description

Two independent centers took part in this study, both representing high-volume departments for septic and aseptic revision arthroplasty. Center A is a university hospital specialized in complex revision in case of failed TKA or total hip arthroplasty (THA). Center B is a hospital specialized in trauma care following work accidents and consecutive posttraumatic joint disorders, including the treatment of any complication occurring after joint arthroplasties, featuring a dedicated unit for septic surgery.

### Treatment algorithms

Prior to revision of the implant, detailed case assessment took place, and treatment options were discussed with an interdisciplinary team as well as the patient. In case of a PJI, the team consisted of the responsible orthopaedic surgeon, a microbiologist, and a pharmacist.

**Fig. 2:**
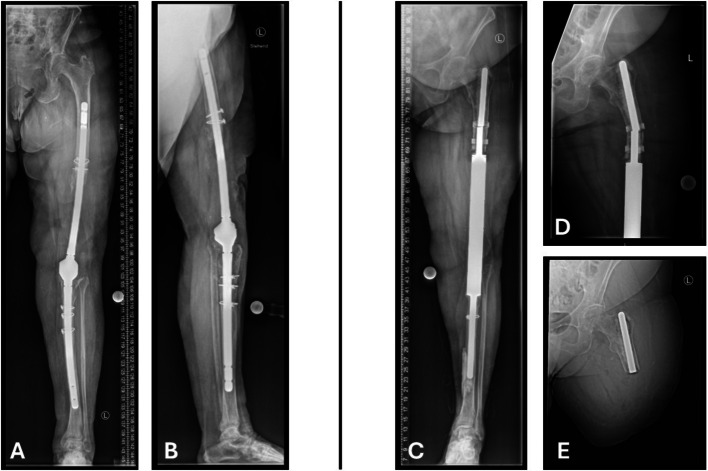
Representative radiographs showing an intact modular knee arthrodesis (MKA) (**A**, **B**) and a failed MKA due to a broken femoral stem (**C**, **D**), necessitating a consecutive proximal transfemoral amputation (**E**)

Surgical treatment included single-, two-, or multi-stage revision depending on the general health status of the patient and extent of infection. Single-stage revision was the preferred option in aseptic cases, while two- and multi-stage options were chosen in most septic cases. Two-stage revision involved an interval of approximately six weeks, extended when clinically indicated, with an antibiotic-loaded cement spacer and systemic antibiotics administered for 12 weeks. Multi-stage revision was defined as any treatment protocol requiring more than two surgical stages before definitive implantation of the modular arthrodesis. Standardized tissue cultures and histopathological samples were obtained in all cases, while implant sonication was performed only at Center A. Four different modular arthrodesis models were in use during the study period (KAM-arthrodesis, Peter Brehm, Weisendorf, Germany; Endo-Model Knee Fusion Nail SK, Waldemar Link, Hamburg, Germany; REVISIO RTM-System, AQ Implants, Ahrensburg, Germany; MUTARS RS arthrodesis, Implantcast, Buxtehude, Germany) and used either a cemented (Endo-Model Link; Mutars RS Implantcast) or cementless (KAM P. Brehm; Revisio RTM AQ Implants) stem fixation. All systems provided stability through the connection module and did not require osseous fusion between the femur and tibia. Representative radiographs are shown in Fig. 2.

Postoperative rehabilitation typically involved six weeks of partial weight bearing, except for cemented implants, which allowed immediate full weight bearing.

### Outcome assessment

The primary endpoint was defined as the overall implant survival. This refers to either a partial (femoral or tibial) or total replacement of the arthrodesis or an AKA. Secondary endpoints were the infection-free and all-cause revision-free survival, rates of consecutive amputations, and the functional outcome and quality of life. The infection-free survival was considered successful according to the Delphi-based International Multidisciplinary Consensus consisting of three features: (1) healed wound without fistula, drainage or pain, indicating infection eradication and no infection recurrence caused by the same organism strain, (2) no subsequent surgical intervention for infection after reimplantation surgery (PJI-revision-free survival) and (3) no occurrence of PJI-related mortality [13]. The revision-free survival refers to any surgical revision of the affected joint (implant replacement, DAIR, wash-out, osteosynthesis, AKA).

Follow-up was conducted through outpatient clinic visits and questionnaires regarding the functional outcome and quality of life. We recorded the lower extremity function score (LEFS) to evaluate the functional outcome as well as the EQ-5D-3L (EuroQol Research Foundation, Rotterdam, Netherlands) for quality of life. Patient-reported outcome measures (PROM) analyses were restricted to patients alive with a retained modular arthrodesis at the latest follow-up. Patients who had undergone above-the-knee amputation, had died before follow-up assessment, or had no available PROM data were excluded from PROM analyses. The overall PROM completion rate was 30 out of 85 cases (35.3%) and 30 out of 60 (50.0%) of all eligible cases (arthrodesis still in situ, patient alive).

The LEFS results in a number ranging from 0 to 80, with 80 being the best possible result. It refers to overall daily activity with the affected lower limb only through questions to the patient.

The EQ-5D-3L (© EuroQol Research Foundation. EQ-5D™ is a trademark of the EuroQol Research Foundation.) is a validated score for assessing quality of life through 5 items on overall mobility, self-care, activity, pain, and anxiety. Each answer has three levels between none and extreme problems/anxiety/pain. The results are then put into relation to the country’s healthy population and given as an index between 0.0 and 1.0, with 1.0 being the best possible result [14]. Additionally, the overall health status of the patient is assessed by asking the subject to give his or her health as a value between 0 and 100, resulting in the EQ-visual analogue scale (EQ-VAS) for general health.

### Ethics and statistical analysis

The study was approved by the local institution’s Ethics Committee (reference no. 2024-645-S-NP) and was conducted in accordance with the Helsinki Declaration. Written patient consent was obtained in advance.

Normally distributed variables are given as mean and standard deviation (SD), non-normally distributed variables as median and interquartile range (IQR). The Shapiro–Wilk Test was used to assess whether the variables followed a normal distribution. Between-group comparisons for continuous variables were conducted using the Student’s t-test or the Mann–Whitney U-test, as appropriate. Categorical variables are presented as frequencies and percentages and compared using the χ^2^ test. A *p*-value ≤ 0.05 was considered statistically significant. Survival analysis was done using Kaplan–Meier survival statistics. Log-rank-test and Cox regression were used for survival comparisons. Hazard ratios (HR) are given with 95% confidence intervals in brackets. Youden’s index was used as an exploratory method to define a cut-off for the number of previous surgeries.

Given the limited number of implant failures (*n* = 25), only four clinically relevant variables were included in the multivariable model to reduce the risk of overfitting. Variable selection was based on clinical relevance and a priori considerations rather than solely on univariable statistical significance.

Statistical analysis and generation of all figures were carried out using IBM SPSS Statistics for Windows, version 31.0 (IBM Corporation, Armonk, New York, USA).

## Results

### Demographics

85 cases were included in the following study population (Center A: *n* = 43/Center B: *n* = 42). The mean (SD) and median (IQR) follow-up were 52.0 (49.4) and 35.0 (46.0) months, respectively. Cases without treatment failure were followed for at least 12 months. Detailed patients’ demographics are demonstrated in Table 1.

**Table 1:** Patients’ demographics and implant data

		**Total (*n* = 85)**	**Center A** **(*n* = 43)**	**Center B** **(*n* = 42)**	***p*-value**
Age in years (median; IQR)		69.0 (15.5)	70.0 (17.0)	68.5 (14.5)	0.266
Sex (male/female; *n*; female share in %)		39/46 (54.1)	15/28 (65.1)	24/18 (42.9)	**0.039**
Charlson Comorbidity Score (median; IQR)		4.0 (3.0)	4.0 (3.0)	4.0 (3.0)	0.686
BMI in kg/m^2^ (mean; SD)		31.3 (7.6)	29.1 (5.4)	33.0 (8.6)	**0.029**
Follow-up in months (mean; SD)		52.0 (49.4)	46.7 (33.1)	57.3 (61.8)	0.329
Follow-up ≥ 24 months; failed cases excluded (*n*; %)		48/60 (80.0)	26/35 (74.3)	22/25 (88.0)	0.406
Follow-up ≥ 60 months; failed cases excluded (*n*; %)		25/60 (41.7)	12/35 (34.3)	13/25 (52.0)	0.193
ASA score (*n*; %)	ASA I	1 (1.2)	1 (2.3)	-	0.320
	ASA II	34 (40.0)	19 (44.2)	15 (35.7)	0.425
	ASA III	48 (56.5)	23 (53.5)	25 (59.5)	0.575
	ASA IV	2 (2.4)	-	2 (4.8)	0.148
Indication for arthrodesis	PJI	59 (69.4)	26 (60.5)	33 (78.6)	0.095
	Aseptic loosening	9 (10.6)	2 (4.7)	7 (16.7)	0.072
	Irreparable failure of extensor mechanism	6 (7.1)	5 (11.6)	1 (2.4)	0.096
	Painful TKA without structural correlates	8 (9.4)	8 (18.6)	-	**0.003**
	Mechanical failure of TKA	1 (1.2)	1 (2.3)	-	0.320
	Periprosthetic fracture	2 (2.4)	1 (2.3)	1 (2.4)	0.972
Deficient extensor mechanism at time of arthrodesis (*n*; %)		59 (69.4)	29 (67.4)	30 (71.4)	0.690
Type of surgical therapy (*n*; %)	Single-stage revision	26 (30.6)	13 (30.2)	13 (31.0)	0.943
	Two-stage revision	22 (25.9)	17 (39.5)	5 (11.9)	**0.004**
	Multi-stage revision	37 (43.5)	13 (30.2)	24 (57.1)	**0.012**
Implant type (*n*; %)	KAM-arthrodesis (*P. Brehm*)	70 (82.4)	33 (76.7)	37 (88.1)	0.170
	MUTARS RS (*Implantcast*)	6 (7.1)	5 (11.6)	1 (2.4)	0.096
	Revisio RTM (*AQ Implants*)	7 (8.2)	5 (11.6)	2 (4.8)	0.250
	Endo-Model (*W. Link*)	2 (2.4)	-	2 (4.8)	0.148
Type of fixation (*n*; %)	Cementless	76 (89.4)	38 (88.4)	38 (90.5)	
	Cemented	9 (10.6)	5 (11.6)	4 (9.5)	
Duration of surgery in minutes (median; IQR)		144.0 (68.7)	134.5 (69.5)	147.5 (52.0)	0.478
No. of prior surgeries (mean; SD)		4.0 (3.0)	4.5 (3.75)	4.0 (4.0)	0.201
Time from primary TKA to arthrodesis in months (median; IQR)		54.0 (102.0)	51.5 (105.5)	60.0 (85.0)	0.960
Prior PJI in history (*n*; %)		64 (75.3)	30 (69.8)	34 (81.0)	0.232

Bold values indicate a significant difference (*p* < 0.05). IQR = interquartile range; SD = standard deviation; ASA = American Society of Anesthesiologists; TKA = total knee arthroplasty; PJI = periprosthetic joint infection

### Outcome

Sixty out of eighty-five patients (70.6%) retained their MKA until the latest follow-up. Two- and five-year implant survival rates (95% CI; number at risk) following Kaplan–Meier analysis were 81.9% (73.7–90.1; 65) and 67.0% (55.2–78.8; 27). Consecutively, 17 revisions of the arthrodesis involving the partial or complete replacement of the implant and 8 AKAs had to be performed. Reasons for failure were recurring PJI in 8 cases (32.0%), aseptic loosening in 10 cases (40.0%), persisting pain in 3 cases (12.0%), implant failure in 3 cases (12.0%), and a periprosthetic fracture in 1 case (4.0%).

The crude revision-free survival proportion was 63.5% (54/85). In addition to the implant revisions and amputations, 2 (6.5%) osteosyntheses for periprosthetic fractures and 4 (12.9%) DAIR procedures (debridement, antibiotics, and implant retention) for recurring PJI were performed. Regarding the crude infection-free survival proportion at the latest follow-up after initial PJI, 47 out of 59 cases (79.6%) remained without any signs of recurring PJI. In comparison, 1 aseptic arthrodesis had to be revised for PJI (1/26; 3.8%).

Single-stage revision was the preferred type of treatment for aseptic cases (65.4%; 17/26), followed by two-stage (23.1%; 6/26) and multi-stage revision (11.5%; 3/26). For septic cases, multi-stage revision represented the preferred treatment option (57.6%; 34/59), followed by two-stage (27.1%; 16/59) and single-stage revision (15.3%; 9/59).

**Fig. 3:**
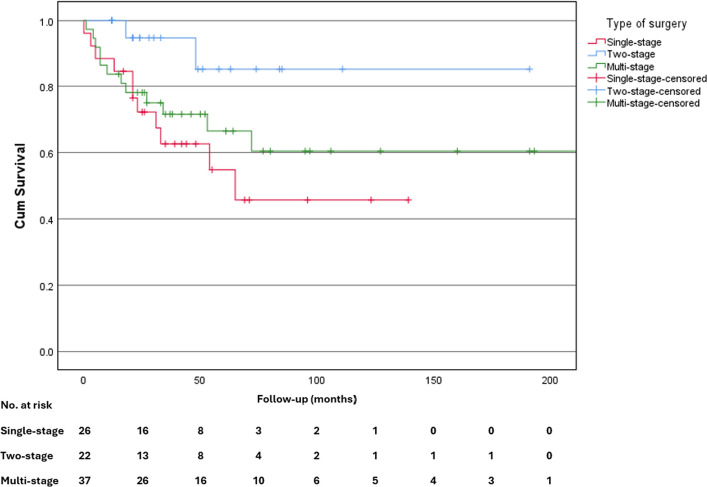
Kaplan–Meier analysis on overall implant survival. (= partial/total replacement of arthrodesis or above the knee amputation) comparing the type of surgery. Two-stage revision provided significantly better outcomes in comparison to single-stage revision (HR: 0.2 (95% CI 0.05–0.98); *p* = 0.048). There was no significant difference between single- and multi-stage revision (HR: 0.7 (95% CI 0.3–1.6); *p* = 0.395) and two- and multi-stage revision (HR: 3.6 (95% CI 0.8–15.9); *p* = 0.097)

In univariate analysis, two-stage approach provided the highest crude implant survival proportion of 90.9% (20/22), followed by multi-stage (67.6%; 25/37; *p* = 0.042) and single-stage revision (57.7%; 15/26; *p* = 0.010) (Fig. 3). Center A showed a numerically higher implant survival than center B (81.4%; 35/43 vs. 59.5%; 25/42; *p* = 0.050). Septic arthrodesis did not differ significantly from aseptic indications regarding the overall implant survival at latest follow-up (42/59; 71.2% vs. 18/26; 69.2%; *p* = 0.855). When a multivariable Cox regression analysis including surgical therapy, center, initial indication, and number of prior surgeries was performed, the previously observed center effect was no longer apparent. A further analysis of potential risk factors is demonstrated in Table 2.

**Table 2:** Univariable/multivariable Cox regression for potential risk factors for overall implant survival

		**Implant survival proportion (*n*; %)**	**Univariate Hazard ratio**	**95% CI**	***p*-value**	**Multivariate adjusted Hazard ratio**	**95% CI**	***p*-value**
**Nr. of prior surgeries**	≤ 4	35/42 (83.3)	Ref	-	-	Ref	-	-
	≥ 5	21/38 (55.3)	3.2	1.3–7.8	**0.006**	4.5	1.7–11.6	**0.002**
	Incremental increase by 1	-	1.1	0.9–1.3	0.137	-	-	-
**Center**	Center A	35/43 (81.4)	Ref	-	-	Ref	-	-
	Center B	25/42 (59.5)	2.3	1.0–5.2	0.050	1.7	0.7–4.3	0.244
**Indication for arthrodesis**	PJI	42/59 (71.2)	Ref	-	-	Ref	-	-
	All aseptic indications	18/26 (69.2)	1.0	0.4–2.1	0.855	0.6	0.2–1.7	0.355
	Aseptic loosening	7/9 (77.8)	0.5	0.1–2.3	0.390	-		
	Periprosthetic fracture	2/2 (100.0)	-	-	-	-	-	-
	Extensor mechanism failure	4/6 (66.7)	1.2	0.3–5.1	0.824	-	-	-
	Failure of prosthesis	1/1 (100.0)	-	-	-	-	-	-
	Painful prosthesis	4/8 (50.0)	1.5	0.5–4.4	0.491	-	-	-
**Type of surgical therapy**	Single-stage	15/26 (57.7)	Ref	-	-	Ref	-	-
	Two-stage	20/22 (90.9)	0.2	0.0–1.0	**0.048**	0.13	0.0–0.7	**0.02**
	Multi-stage	25/37 (67.6)	0.7	0.3–1.6	0.395	0.3	0.1–0.8	**0.01**
**Sex**	Male	28/39 (71.8)	Ref	-	-			
	Female	32/46 (69.6)	0.9	0.4–2.2	0.950			
**Age**	< 65 years	30/46 (65.2)	Ref	-	-			
	> 65 years	30/39 (76.9)	0.8	0.3–1.7	0.502			
**Obesity**	BMI < 30	34/48 (70.8)	Ref	-	-			
	BMI > 30	25/36 (69.4)	0.8	0.4–1.9	0.720			
**ASA-score**	1	1/1 (100.0)	-	-	-			
	2	20/34 (58.8)	Ref	-	-			
	3	38/48 (79.2)	0.5	0.2–1.1	0.093			
	4	1/2 (50.0)	2.2	0.3–17.1	0.444			
**Prior type of TKA**	Primary TKA	14/16 (87.5)	Ref	-	-			
	Revision TKA	40/60 (66.7)	2.7	0.6–11.5	0.183			
	Distal femoral replacement	6/9 (66.7)	3.1	0.5–18.7	0.214			

*Note:* Overall implant survival was defined as freedom from partial/total replacement of arthrodesis or above-the-knee amputation. Because only 25 implant failures occurred, the multivariable Cox model was restricted to four prespecified clinically relevant variables (number of prior surgeries, centre, indication for arthrodesis, and surgical strategy) to reduce the risk of overfitting. Bold values indicate a significant difference (*p* < 0.05)

### Amputations

An AKA had to be performed in 3 out of 59 cases (5.1%) of initially septic indications. In all 3 cases, the amputation was indicated by recurring, uncontrollable PJI. In comparison, of the patients with initially aseptic indications, 5 out of 26 cases (19.2%) ultimately underwent an AKA (*p* = 0.042) (Table 3). The reasons for amputation were a PJI in 1 case (20.0%), a non-reconstructable periprosthetic fracture in 1 case (20.0%), and persisting pain without any underlying structural causes in 3 cases (60.0%).

**Table 3:** Outcomes stratified by indication for modular knee arthrodesis

Indication for arthrodesis	*n*	Implant failure	All-cause revisions	AKA
PJI (*n*; %)	59	17 (28.8)	23 (39.0)	3 (5.1)
Aseptic loosening (*n*; %)	9	2 (22.2)	2 (22.2)	0
Periprosthetic fracture (*n*; %)	2	0	0	0
Extensor mechanism failure (*n*; %)	6	2 (33.3)	2 (33.3)	1 (16.7)
Failure of prosthesis (*n*; %)	1	0	0	0
Painful TKA without structural correlate (*n*; %)	8	4 (50.0)	4 (50.0)	4 (50.0)
All aseptic indications	26	8 (30.8)	8 (30.8)	5 (19.2)
All aseptic indications excl. painful TKA	18	4 (22.2)	4 (22.2)	1 (5.6)
Total	85	25 (29.4)	31 (36.5)	8 (9.4)

*Note:* Implant failure = partial/total replacement of arthrodesis or AKA; AKA = above-the-knee amputation; PJI = periprosthetic joint infection; TKA = total knee arthroplasty

### Functional outcome and quality of life

**Fig. 4:**
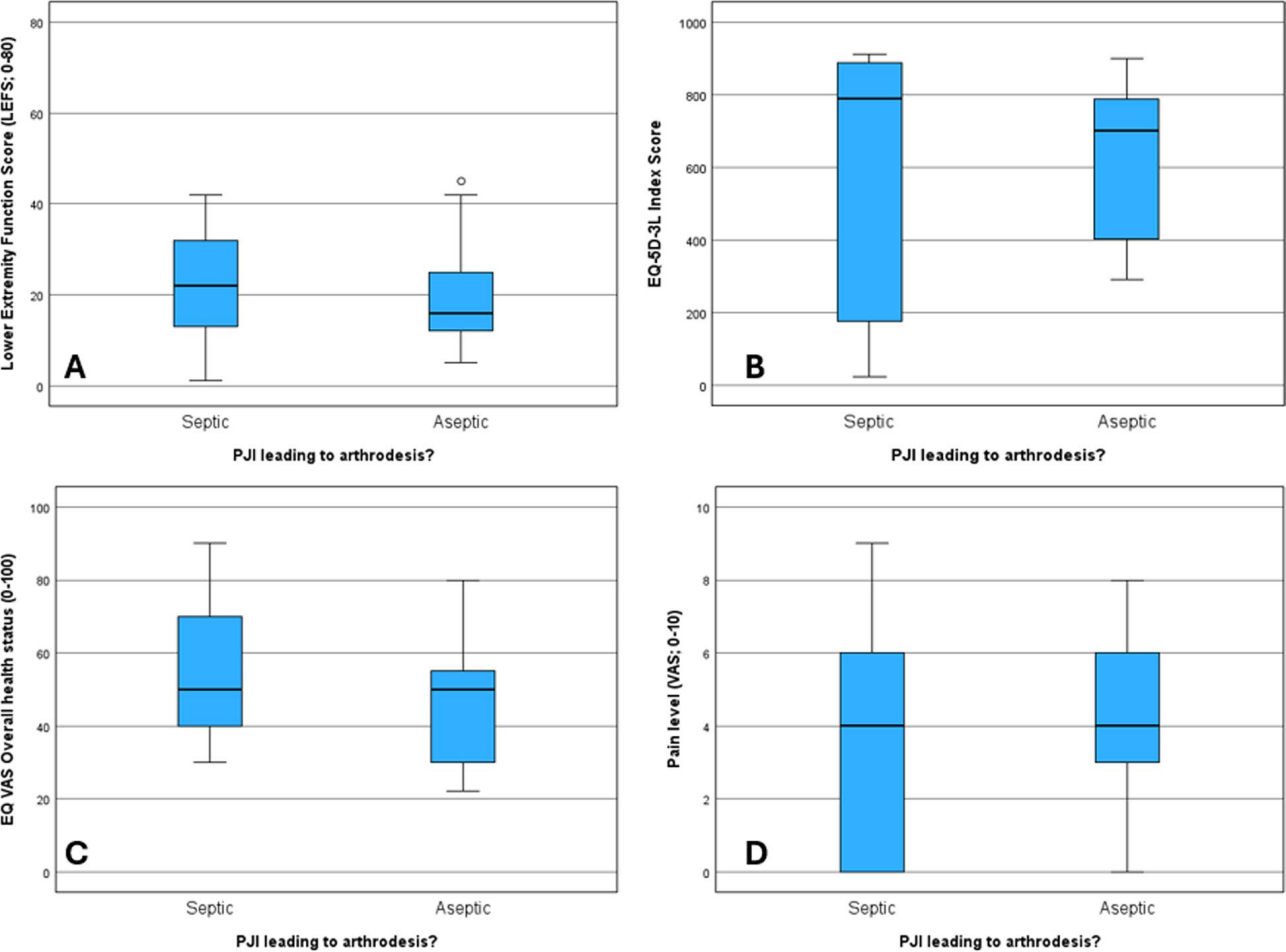
Comparison between septic and aseptic arthrodesis regarding the functional outcome, quality of life and overall health status. A mean (SD) Lower Extremity Function Score (LEFS): septic: 22.7 (12.1)/aseptic: 20.0 (12.1), *p* = 0.542; (**B**) mean (SD) EQ-5D-3L index score: septic: 0.595 (0.341)/0.635 (0.230), *p* = 0.711; (**C**) mean (SD) EQ VAS overall health status: septic: 54.9 (18.1)/45.9 (17.4), *p* = 0.183; (**D**) mean (SD) pain level (visual analogue scale 0–10): septic 3.7 (3.1)/4.2 (2.4), *p* = 0.621

The mean (SD) LEFS was 21.6 (12.0) out of 80 possible points (27.0%). Regarding the general health status, the mean (SD) EQ-5D-3L index score was 0.612 (0.296). The mean (SD) EQ VAS was 51.0 (18.1). There were no significant differences regarding potential factors influencing the functional outcome or general health status. Detailed results are demonstrated in Fig. 4 and Table 4.

**Table 4:** Comparison of potential risk factors for functional outcome, quality of life, general health status, and pain level after modular knee arthrodesis implantation

		**LEFS** (mean; SD)	***p*-value**	**EQ-5D-3L** (mean; SD)	***p*-value**	**EQ VAS** (mean; SD)	***p*-value**	**VAS** (mean; SD)	***p*-value**
**Overall**		21.6 (12.0)		0.612 (0.296)		51.0 (18.1)		3.9 (2.8)	
**Sex**			0.181		0.179		0.859		0.245
	Male	24.8 (12.6)		0.691 (0.263)		50.4 (16.3)		3.3 (2.6)	
	Female	18.9 (11.2)		0.546 (0.314)		51.6 (20.1)		4.5 (3.0)	
**Age**			0.347		0.868		0.455		0.501
	< 65 years	23.3 (12.2)		0.619 (0.312)		53.1 (19.5)		4.2 (2.7)	
	> 65 years	19.2 (11.7)		0.601 (0.285)		47.9 (16.0)		3.5 (3.1)	
**Presence of PJI**			0.542		0.711		0.183		0.621
	Septic	22.7 (12.1)		0.595 (0.341)		54.9 (18.1)		3.7 (3.1)	
	Aseptic	20.0 (12.1)		0.635 (0.230)		45.9 (17.4)		4.2 (2.4)	
**Type of surgery**			0.657		0.438		0.670		0.646
	Single-stage	19.2 (13.2)		0.598 (0.297)		51.9 (16.1)		3.5 (3.1)	
	Two-stage	22.2 (12.3)		0.528 (0.311)		46.6 (21.0)		4.7 (2.5)	
	Multi-stage	23.9 (10.8)		0.704 (0.286)		54.0 (18.5)		3.8 (2.9)	
**Preoperative extensor mechanism**			0.273		0.752		0.222		0.819
	Intact	17.1 (13.0)		0.580 (0.286)		59.2 (20.6)		3.7 (3.0)	
	Insufficient	22.9 (11.7)		0.621 (0.304)		49.0 (17.3)		4.0 (2.8)	
**ASA-score**			0.313		0.908		0.378		0.741
	ASA II	25.1 (14.6)		0.614 (0.336)		54.2 (16.6)		4.2 (2.1)	
	ASA III	19.8 (8.6)		0.617 (0.282)		48.3 (19.2)		3.9 (3.5)	
**Obesity**			0.662		0.127		0.984		0.159
	BMI < 30	20.7 (12.1)		0.686 (0.250)		50.9 (16.0)		3.3 (2.7)	
	BMI > 30	22.6 (12.2)		0.522 (0.331)		51.1 (20.8)		4.7 (2.8)	
**Center**			0.877		0.704		0.452		0.159
	Center A	21.9 (13.1)		0.633 (0.293)		53.7 (16.9)		4.7 (2.1)	
	Center B	21.3 (11.3)		0.592 (0.307)		48.6 (19.3)		3.3 (3.2)	

*Note:* Functional outcome was assessed using the Lower Extremity Functional Scale (LEFS; 0–80 points, with 80 indicating the best outcome). Overall quality of life was assessed using the EQ-5D-3L index score (0.0–1.0, with 1.0 indicating the best outcome). General health status was assessed using the EQ VAS (0–100, with 100 indicating the best outcome). Pain level was assessed using the Visual Analogue Scale (VAS; 0–10, with 10 indicating the most severe pain)

## Discussion

The key finding of this study is the high rate of failure of MKA following failed TKA, which was associated with inferior functional outcomes and reduced quality of life. Despite its role as a salvage procedure in patients with limited reconstructive options, only 60 of 85 patients retained their MKA until the latest follow-up, and more than one third of patients required revision surgery or amputation during the follow-up period.

### Modular knee arthrodesis as a viable PJI treatment?

For many surgeons, an MKA is used as a limb-sparing treatment option for uncontrollable PJI with the rationale that removal of the prosthetic articulation and reduction of the periprosthetic dead space may facilitate infection control. Previous studies have reported widely varying results regarding infection eradication and revision-free survival. Schnetz et al. have found reinfection and revision rates of 11.5% (6/52), respectively [15]. Hungerer et al. reported a reinfection rate of 21.8% (12/55) and a revision rate of 36.4% (20/55) [8]. In a systematic review by Low et al., the overall reinfection rate was 17.2%, and the revision rate was 18.6% in a cohort of 1034 included knee arthrodesis cases [11]. While about a third of all our patients required revision of their arthrodesis irrespective of a septic or aseptic indication, the consecutive infection rate was 20.3% (12/59) and 3.8% (1/26) (*p* = 0.188), respectively. This is in line with previous results for revision TKA when septic surgery results in consecutively elevated reinfection rates [16]. MKA therefore seems to provide (similarly moderate) success rates in septic and aseptic cases. When comparing the results to “conventional arthrodesis” for PJI treatment relying on osseous fusion through an external fixation, re-infection rates are reported to be at 5.4% with a significant benefit over intramedullary devices (10.6%; *p* = 0.009) [17]. However, not all revisions following TKA allow for osseous fusion when distinct osseous defects are present.

### High amputation rates, particularly following painful TKAs

One of the most striking observations of this study is the significantly higher rate of AKAs among patients with initially aseptic indications compared to those treated for PJI. Contrary to the commonly held assumption that septic cases are more likely to require amputation, this study shows that aseptic cases, particularly those with painful TKA without identifiable structural pathology before arthrodesis, carry an unexpectedly high risk. This finding suggests that the indication “painful TKA” may represent a distinct and complex subgroup in which chronic pain syndromes, soft-tissue compromise, or neuropathic components may persist despite technically successful implantation, and all treatment options must be discussed meticulously prior to surgery. These findings raise the question whether modular knee arthrodesis is an appropriate treatment option for patients with painful TKA in the absence of identifiable structural pathology and warrant further investigation of this specific subgroup. The overall amputation rate following MKA was slightly, but non-significantly higher than previously reported by a systematic review by Low et al., who found a mean AKA rate of 7.7% (58/752) within 20 studies [11] (*p* = 0.585). A possible explanation for this difference might be the inclusion of painful TKAs as an indication for MKA. Hungerer et al., on the other hand, reported similar results with a consecutive amputation rate of 10.9% [8].

### Impact of surgical history: extensive prior revisions as a major risk factor

The number of previous surgeries emerged as a strong predictor of failure. Patients with five or more prior procedures had a significantly higher risk of arthrodesis failure, with the hazard ratio tripling compared to those with fewer operations. This finding aligns with previous literature reporting that each additional revision surgery nearly doubles the risk of subsequent failure in revision TKA [18, 19]. Thus, the surgical “burden” prior to arthrodesis appears to be one of the most decisive variables influencing long-term success. The threshold of five previous surgeries was derived post hoc using Youden’s index and should therefore be considered exploratory.

### Center effects and the influence of case mix

Center A demonstrated a numerically higher implant survival than center B. Both centers represent high-volume hospitals performing revision TKA and especially treating periprosthetic joint infections. The only significant differences were the usage of more multi-stage revisions in center B, which might either impose a risk factor or be a surrogate parameter for more complex cases. Overall, the rate of PJIs treated at center B was slightly higher than at center A. After adjusting for other risk factors, the observed difference between centers disappeared. The disappearance of the center effect after adjustment suggests confounding by case mix rather than a true center-specific treatment effect.

### Influence of treatment strategies

The two-stage approach was associated with the highest implant survival of around 90%, significantly outperforming single-stage revision and showing a non-significant benefit over multi-stage revision. This finding supports widely accepted principles in the treatment of PJI where two-stage revision remains the gold standard. However, this cohort included both septic and aseptic cases, raising an important question: Why did the single-stage approach, which is theoretically optimal for aseptic indications, show inferior outcomes? Despite the differences being non-significant within subgroup analysis, there was still a benefit towards two-stage revision in aseptic and septic cases. Several explanations might be proposed: (1) Patients undergoing single-stage procedures may have poorer systemic conditions that contraindicated multiple surgeries but increase the overall risk for failure, especially in cases of PJI. (2) Hidden infections or culture-negative infections not detected prior to implantation of arthrodesis might be a further explanation for the higher failure rate. Despite the tendency towards improved outcomes after two-stage replacement, results must be interpreted carefully due to the retrospective study design and further investigations are necessary.

### Poor functional outcomes and quality of life, consistent across subgroups

Among patients with available PROM data, functional outcomes were poor, with a mean LEFS of only 21.6/80 (27.0%), consistent with the literature describing knee arthrodesis as functionally limiting despite reliable pain reduction. Gramlich et al., in comparison, reported an overall WOMAC score of 40 for MKA, with 0 being the best function (60%) [20]. Hungerer et al., on the other hand, found similar results with a mean LEFS of 28 out of 80 [8], having a non-significant poorer functional outcome in comparison to AKA patients in their cohort. Interestingly, Schnetz et al. reported contrary results in their study with poorer results for patients undergoing AKA in comparison to MKA cases (WOMAC: AKA 48.5 vs. MKA 35.9). It remains controversial whether an amputation or arthrodesis is the best choice for patients suffering from complex TKA complications. The EQ-5D-3L index (0.612) and EQ VAS (51.0) reflect substantial reductions in health-related quality of life. Notably, none of the analyzed variables, including age, BMI, sex, surgical strategy, or septic vs. aseptic indication, showed significant influence on functional or quality-of-life outcomes. This homogeneity suggests that the arthrodesis itself, rather than patient characteristics or surgical technique, imposes a profound and universal limitation. Several studies have reported general and mental health measures for arthrodesis patients, often comparing MKA and AKA. Trouillez et al. reported poorer SF-36 scores for arthrodesis patients in comparison to AKA (22.0 vs. 33.6) [21], which are further supported by the results from Hungerer et al. (SF-12 physical: MKA: 30 vs. AKA: 36). Overall, all patients had significantly poorer results in comparison to the general population and comparable to the reference group with severe extremity disability [20].

As an additional note, several patients in our cohort explicitly mentioned severe back pain caused by the leg length discrepancy as the major limitation in daily life.

## Limitations

Several limitations of this study should be acknowledged. First, the study’s retrospective design inherently has limitations, including potential biases and data collection challenges. Second, the relatively small overall cohort size limits the statistical power and the generalizability of the findings to a broader patient population, especially when comparing different treatment options. However, it is worth noting that this study benefits from a representative pool of patients, with 85 cases included being the largest cohort on MKA available to date. The rare use of MKA causes the cohort sizes to remain small.

Third, the inclusion of aseptic and septic indications might cause heterogeneity in this study. Nevertheless, the comparison of those indications remains an important part of this study as most previous reports have been solely focusing on knee arthrodesis as a PJI salvage treatment.

Fourth, the absence of preoperative PROM limits the ability to conclude the impact of the arthrodesis on the individual pain and functional levels of the patients. Further, PROM were available in only 50% of eligible survivors and did not include patients who underwent amputation, introducing potential survivorship and responder bias.

Finally, mortality and competing risks were not formally modelled because of the limited number of events and the retrospective design.

## Conclusions

In this highly challenging patient population, modular non-fusion knee arthrodesis provides only moderate implant durability and is associated with poor functional outcomes and reduced quality of life in patients available for PROM assessment. Aseptic indications, particularly painful TKA without mechanical failure, are associated with high amputation rates, while an increasing number of prior surgical interventions markedly elevates the risk of treatment failure. Two-stage revision was associated with superior implant survival; however, given the retrospective design and potential selection bias, causal conclusions cannot be drawn. Further studies should focus on characterizing patient-reported pain phenotypes and further compare MKA with AKA, as well as with additional revision total knee arthroplasty when feasible. These findings underscore the substantial challenges inherent in managing patients with prolonged and complex revision histories and confirm the heightened vulnerability of this population to adverse outcomes.

## Data Availability

The dataset supporting the conclusions of this article has not been published anywhere but can be made available on request.

## References

[1] Kurtz SM, et al. Prosthetic joint infection risk after TKA in the Medicare population. Clin Orthop Relat Res. 2010;468(1):52–6. 19669386 10.1007/s11999-009-1013-5 PMC2795807

[2] Peersman G, et al. Infection in total knee replacement: a retrospective review of 6489 total knee replacements. Clin Orthop Relat Res. 2001;392:15–23. 11716377

[3] Kunutsor SK, et al. Patient-related risk factors for periprosthetic joint infection after total joint arthroplasty: a systematic review and meta-analysis. PLoS ONE. 2016;11(3):e0150866. 26938768 10.1371/journal.pone.0150866 PMC4777569

[4] Springer BD, et al. Infection burden in total hip and knee arthroplasties: an international registry-based perspective. Arthroplast Today. 2017;3(2):137–40. 28695187 10.1016/j.artd.2017.05.003 PMC5485227

[5] Gausden EB, et al. Synchronous periprosthetic joint infections: high mortality, reinfection, and reoperation. J Arthroplasty. 2021;36(10):3556–61. 34088568 10.1016/j.arth.2021.05.010

[6] Kurtz SM, et al. Economic burden of periprosthetic joint infection in the United States. J Arthroplasty. 2012;27(8 Suppl):61-5.e1. 22554729 10.1016/j.arth.2012.02.022

[7] Premkumar A, et al. Projected economic burden of periprosthetic joint infection of the hip and knee in the United States. J Arthroplasty. 2021;36(5):1484-1489.e3. 33422392 10.1016/j.arth.2020.12.005

[8] Hungerer S, et al. Knee arthrodesis versus above-the-knee amputation after septic failure of revision total knee arthroplasty: comparison of functional outcome and complication rates. BMC Musculoskelet Disord. 2017;18(1):443. 29132347 10.1186/s12891-017-1806-8 PMC5683527

[9] Jones RE, Russell RD, Huo MH. Alternatives to revision total knee arthroplasty. J Bone Joint Surg Br. 2012;94(11 Suppl A):137–40. 23118402 10.1302/0301-620X.94B11.30620

[10] Gramlich Y, Parvizi J. Enough is enough: salvage procedures in severe periprosthetic joint infection. Arthroplasty. 2023;5(1):36. 37394449 10.1186/s42836-023-00182-7 PMC10316561

[11] Low J, et al. Transfemoral amputation versus knee arthrodesis for failed total knee replacement: a systematic review of outcomes. Knee. 2024;47:63–80. 38245922 10.1016/j.knee.2023.12.012

[12] McNally M, et al. The EBJIS definition of periprosthetic joint infection. Bone Joint J. 2021;103-b(1):18–25. 33380199 10.1302/0301-620X.103B1.BJJ-2020-1381.R1 PMC7954183

[13] Diaz-Ledezma C, Higuera CA, Parvizi J. Success after treatment of periprosthetic joint infection: a Delphi-based international multidisciplinary consensus. Clin Orthop Relat Res. 2013;471(7):2374–82. 23440616 10.1007/s11999-013-2866-1 PMC3676607

[14] Greiner W, et al. Validating the EQ-5D with time trade off for the German population. Eur J Health Econ. 2005;6(2):124–30. 19787848 10.1007/s10198-004-0264-z

[15] Schnetz M, et al. Above-knee amputation shows higher complication and mortality rates in line with lower functional outcome compared to knee arthrodesis in severe periprosthetic joint infection. Bone Joint J. 2024;106-b(7):669–79. 38946307 10.1302/0301-620X.106B7.BJJ-2023-0978.R2

[16] Lee CR, et al. Risk of infection after septic and aseptic revision total knee arthroplasty: a systematic review. J Bone Joint Surg Am. 2023;105(20):1630–7. 37616387 10.2106/JBJS.23.00361

[17] Balato G, et al. Re-infection rates and clinical outcomes following arthrodesis with intramedullary nail and external fixator for infected knee prosthesis: a systematic review and meta-analysis. BMC Musculoskelet Disord. 2018;19(1):361. 30301462 10.1186/s12891-018-2283-4 PMC6178263

[18] Deere K, et al. How long do revised and multiply revised knee replacements last? A retrospective observational study of the National Joint Registry. Lancet Rheumatol. 2021;3(6):e438–46. 35043097 10.1016/S2665-9913(21)00079-5 PMC7612217

[19] Friedrich MJ, et al. Two-stage knee arthrodesis with a modular intramedullary nail due to septic failure of revision total knee arthroplasty with extensor mechanism deficiency. Knee. 2017;24(5):1240–6. 28622842 10.1016/j.knee.2017.05.019

[20] Gramlich Y, et al. Modular knee arthrodesis secures limb, mobility, improves quality of life, and leads to high infection control in periprosthetic knee infection, when revision knee arthroplasty is not an option. Arch Orthop Trauma Surg. 2021;141(8):1349–60. 33893531 10.1007/s00402-021-03907-y

[21] Trouillez T, et al. Above-the-knee amputation versus knee arthrodesis for revision of infected total knee arthroplasty: recurrent infection rates and functional outcomes of 43 patients at a mean follow-up of 6.7 years. Orthop Traumatol Surg Res. 2021;107(4):102914. 33812091 10.1016/j.otsr.2021.102914

